# Acute myocarditis and multisystem inflammatory emerging disease following SARS-CoV-2 infection in critically ill children

**DOI:** 10.1186/s13613-020-00690-8

**Published:** 2020-06-01

**Authors:** Marion Grimaud, Julie Starck, Michael Levy, Clémence Marais, Judith Chareyre, Diala Khraiche, Marianne Leruez-Ville, Pierre Quartier, Pierre Louis Léger, Guillaume Geslain, Nada Semaan, Florence Moulin, Matthieu Bendavid, Sandrine Jean, Géraldine Poncelet, Sylvain Renolleau, Mehdi Oualha

**Affiliations:** 1 Pediatric Intensive Care Unit, Necker-Enfants-Malades University Hospital, Assistance Publique-Hôpitaux de Paris, Paris, France; 2 Pediatric and Neonatal Intensive Care Unit, Armand-Trousseau University Hospital, Assistance Publique-Hôpitaux de Paris, Paris, France; 3 Pediatric Intensive Care Unit, Robert Debré University Hospital, Assistance Publique-Hôpitaux de Paris, Université de Paris, Paris, France; 4 Pediatric and Neonatal Intensive Care Unit, Kremlin-Bicêtre University Hospital, Assistance Publique-Hôpitaux de Paris, Paris, France; 5 M3C-Necker, Congenital and Pediatric Cardiology, Necker-Enfants-Malades University Hospital, Assistance Publique-Hôpitaux de Paris, Paris, France; 6 Laboratoire de Virologie, Paris University, EA 7328, Paris, France; 7 Paediatric Hematology-Immunology and Rheumatology Unit, Reference center for Rheumatic, AutoImmune and Systemic diseases in children (RAISE), Necker-Enfants Malades University Hospital, Assistance Publique-Hôpitaux de Paris, IMAGINE Institute, Université de Paris, Paris, France; 8 Pediatric and Neonatal Intensive Care unit, Armand-Trousseau University Hospital, Assistance Publique-Hôpitaux de Paris, Sorbonne University, Paris, France; 9 Pediatric Intensive Care Unit, Necker-Enfants-Malades University Hospital, Assistance Publique-Hôpitaux de Paris, Paris University, EA7323, 75006 Paris, France

**Keywords:** Shock, Children, Acute myocarditis, Multisystem inflammatory syndrome, SARS-CoV-2

## Abstract

**Background:**

A recent increase in children admitted with hypotensive shock and fever in the context of the COVID-19 outbreak requires an urgent characterization and assessment of the involvement of SARS-CoV-2 infection. This is a case series performed at 4 academic tertiary care centers in Paris of all the children admitted to the pediatric intensive care unit (PICU) with shock, fever and suspected SARS-CoV-2 infection between April 15th and April 27th, 2020.

**Results:**

20 critically ill children admitted for shock had an acute myocarditis (left ventricular ejection fraction, 35% (25–55); troponin, 269 ng/mL (31–4607)), and arterial hypotension with mainly vasoplegic clinical presentation. The first symptoms before PICU admission were intense abdominal pain and fever for 6 days (1–10). All children had highly elevated C-reactive protein (> 94 mg/L) and procalcitonin (> 1.6 ng/mL) without microbial cause. At least one feature of Kawasaki disease was found in all children (fever, *n* = 20, skin rash, *n* = 10; conjunctivitis, *n* = 6; cheilitis, *n* = 5; adenitis, *n* = 2), but none had the typical form. SARS-CoV-2 PCR and serology were positive for 10 and 15 children, respectively. One child had both negative SARS-CoV-2 PCR and serology, but had a typical SARS-CoV-2 chest tomography scan. All children but one needed an inotropic/vasoactive drug support (epinephrine, *n* = 12; milrinone, *n* = 10; dobutamine, *n* = 6, norepinephrine, *n* = 4) and 8 were intubated. All children received intravenous immunoglobulin (2 g per kilogram) with adjuvant corticosteroids (*n* = 2), IL 1 receptor antagonist (*n* = 1) or a monoclonal antibody against IL-6 receptor (*n* = 1). All children survived and were afebrile with a full left ventricular function recovery at PICU discharge.

**Conclusions:**

Acute myocarditis with intense systemic inflammation and atypical Kawasaki disease is an emerging severe pediatric disease following SARS-CoV-2 infection. Early recognition of this disease is needed and referral to an expert center is recommended. A delayed and inappropriate host immunological response is suspected. While underlying mechanisms remain unclear, further investigations are required to target an optimal treatment.

## Introduction

In contrast to adults, severe pediatric cases of the 2019 novel coronavirus (SARS-CoV-2) infections are known to be rare [1, 2]. From the beginning of the European epidemic and until mid-April, most of the critically ill children with SARS-CoV-2 infection presented with respiratory failure and high proportion of co-morbidities [3]. Unexpectedly, within the last 2 weeks, we observed an outbreak of shock cases with high systemic inflammation and myocarditis, suspected to be associated with SARS-CoV-2 infection in four pediatric intensive care units (PICU) in the Paris region. Simultaneously, both UK and French health authorities have alerted pediatricians about an unusual number of children admitted to a PICU with mixed shock and Kawasaki disease. Acute heart failure and myocarditis associated with SARS-CoV-2 has not been reported in children yet, although previously described in adults [4–7]. Here, we aim to describe the characteristics, management and time course of 20 critically ill children admitted to the PICU with cardiogenic shock secondary to acute myocarditis and suspected SARS-CoV-2 infection.

## Methods

This study is a retrospective, observational study performed at 4 academic tertiary care centers in Paris. It was conducted according to the local ethics committee and oral informed consent was obtained from the parents of the children studied. All patients aged less than 18 and admitted to the PICU with shock, fever and a suspected SARS-CoV-2 infection between April 15th and April 27th, 2020 were included in the study population.

Acute myocarditis was defined with the following criteria: elevated troponin, ST segment elevation or depression on electrocardiogram, regional wall motion abnormalities with decreased left ventricular function on echocardiography [8]. Shock was defined as tachycardia and one of the following signs: arterial systemic hypotension, cold extremity, decrease peripheral pulse, capillary refill time > 3 s, oliguria or arterial blood lactate > 2 mmol/L [9]. In addition to usual laboratory investigations, chest X-rays, transthoracic echocardiography, electrocardiogram, troponin, brain natriuretic peptide and lactate measurements were performed. Standard microbiological screening was performed. Usual etiologies of acute myocarditis were systematically screened including the testing of a large panel of non-SARS-CoV-2 viruses in blood, feces and nasopharyngeal swabs. SARS-CoV-2 was diagnosed using polymerase chain reaction (PCR) on nasopharyngeal swabs and/or feces (Emag, BioMérieux™, Marcy-l’Etoile, France; SARS-CoV-2 R-Gene, BioMérieux™). SARS-CoV-2 antibodies (IgG and IgA) were measured using a commercial ELISA assay (anti-SARS-CoV-2 IgA and IgG, EuroImmun™, Bussy-Saint-Martin, France).

Data were extracted from the electronic medical records and de-identified. Descriptive statistics were obtained for all study variables. Continuous data were expressed as median and range values. Categorical data were expressed as proportions (%) Additional file 1: Table S1.

## Results

**Fig. 1:**
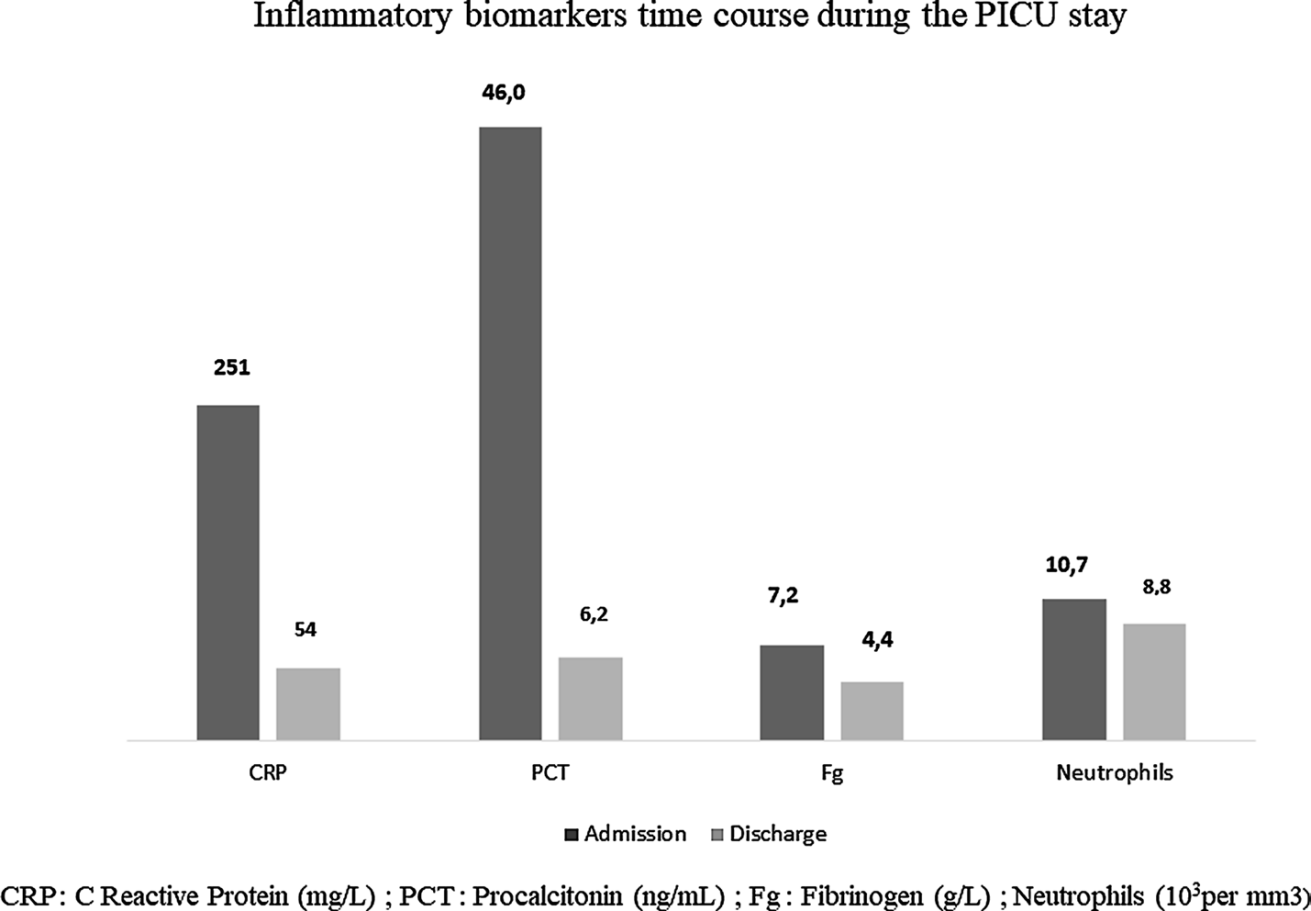
Inflammatory biomarkers time course during the PICU stay. Laboratory values of CRP, PCT, and neutrophil count at PICU admission (black histograms) and discharge (grey histograms)

All the children were hypotensive and presented with a major systemic inflammation and an acute myocarditis. The demographic data, clinical, biological and echocardiographic features of the 20 children are summarized in Table 1. Details of each case are provided in Table S1. No co-morbidity was identified, and no history of previous SARS-CoV-2 symptomatic infection was reported. First, all presented severe abdominal pain, vomiting and fever. The length of the PICU stay for the 15 children already discharged was 4 days (1–8). In addition to acute myocardial systolic dysfunction, all children were hypotensive and 15 showed clinical signs of vasoplegia (warm extremity and immediate capillary refill time). The criteria mentioned before for the myocarditis definition were observed in all patients. Pericardial effusion was observed in 4 children. Coronary arteries were not dilated at this early stage. All children but one received an inotropic support for a median duration of 3 days (1–7). No patient required veno-arterial extracorporeal membrane oxygenation. None of the children had a primary respiratory failure. Invasive mechanical ventilation (*n* = 8) lasted 5 days (1–7). 14 children had transient acute renal failure with no need for renal replacement therapy. None of the patients had sufficient criteria for typical Kawasaki disease, but all had a prolonged fever and half of them a skin rash. Neither bacterial nor non-SARS-CoV-2 viral infections were identified. All children received intravenous immunoglobulin (2 g per kilogram) within the first 2 days of their PICU stay and 18 were thereafter afebrile. Two patients received corticosteroids and two others IL1 receptor antagonist (*n* = 1) or a monoclonal antibody against IL-6 receptor (*n* = 1). All children survived at this stage of the disease and were discharged from the PICU with full left ventricular systolic function recovery and a substantial decrease of inflammatory biomarkers (Fig. 1). SARS-CoV-2 was positive on nasopharyngeal swabs and feces in 10 and 2 children, respectively. SARS-CoV-2 antibodies were positive for all the patients screened at this stage (*n* = 15). Nineteen of the 20 included patients had identified SARS-CoV-2 infection on PCR and/or by serology. The remaining patient had negative SARS-CoV-2 PCR and serology, but his chest computed tomography scan was typical of SARS-CoV-2 infection.

**Table 1:** Clinical, biological and hemodynamic characteristics

	Value
Age, years median (range)	10 (2.9–15)
Sex, no. (%)	
Male	10 (50)
Female	10 (50)
Admission delay from first symptoms, days, median (range)	6 (1–10)
PELOD-2 score at admission, median (range)	10 (10–22)
Clinical description	
Fever, no. (%)	20 (100)
Abdominal pain, no. (%)	20 (100)
Skin rash, no. (%)	10 (50)
Conjunctivitis, no. (%)	6 (30)
Cheilitis, no. (%)	5 (25)
Adenitis (diameter > 1.5 cm), no. (%)	2 (10)
Glasgow Coma Scale, median (range)	15 (4–15)
Hemodynamic features	
Tachycardia, no. (%)	20 (100)
Systemic arterial hypotension, no. (%)	20 (100)
Left ventricular ejection fraction, median (range), %	35 (25–55)
Laboratory values, median (range)^e^	
Troponin, ng/mL	269 (31–4607)
Brain natriuretic peptide, pmol/L^a^	3405 (179–19013)
Lactate, mmol/L	3.6 (1–8,1)
Creatinine clearance, mL/min/1,73 m^2^	97 (16–170)
Albumin, g/L	21 (17–26)
Sodium, mmol/L	131 (122–139)
Alanine aminotransferase, IU/L	27 (6–163)
Platelets count, per mm^3^	210, 000 (93,000–403,000)
Neutrophil count, per mm^3^	10955 (1500–34200)
Lymphocyte count, per mm^3^	1150 (380–7200)
C-reactive protein, mg/L	251 (94–458)
Procalcitonin, ng/mL^b^	46 (1,6–448)
Fibrinogen, g/L^c^	7,2 (3,9–9)
Inotropes and vasoactive drugs, no. (%)	19 (95)
Epinephrine, no. (%)	12 (60)
Median (range), µg/kg/min	0.13 (0,1–0.5)
Milrinone, no. (%)	10 (50)
Median (range), µg/kg/min	0.45 (0.3–0.6)
Dobutamine, no. (%)	6 (30)
Median (range), µg/kg/min	7.5 (5–15)
Norepinephrine, no. (%)	4 (20)
Median (range), µg/kg/min	0.55 (0,2–1.2)
Mechanical ventilation	
Non-invasive ventilation, no. (%)	11 (55)
Invasive ventilation, no. (%)	8 (40)
High-flow nasal oxygen, no. (%)	1 (5)

^a^Available for 15 patients

^b^Available for 16 patients

^c^Available for 19 patients

^d^Highest dosing during the PICU stay

^e^Worst values within the first 24 h of PICU stay

*PELOD-2* Pediatric Logistic Organ Dysfunction 2 score

## Discussion

We describe here the first case series of acute myocarditis and major systemic inflammation following SARS-CoV-2 infection in 20 critically ill children. Very similar clinical and biological presentations, echocardiographic features and time course were observed.

Given the results of SARS-CoV-2 screening antibodies, the association of such a presentation and a SARS-CoV-2 infection is highly likely. The phenotype of our patients differs from those infected by SARS-CoV-2 and previously reported in literature: they are older, have no co-morbidity nor any respiratory failure [1, 2]. The time of occurrence of this emerging disease (delayed by 4 weeks after the beginning of the French lockdown) and the remarkably high rate of IgG and IgA identification strongly suggest a post-viral immunological reaction impacting the myocardium [10]. Besides, the dramatic cardiac function improvement as well as the significant decrease of inflammatory biomarkers following intravenous immunoglobulin reinforces the hypothesis of a SARS-CoV-2 post-infective disease.

In this series, COVID-19 acute myocarditises are less severe than those usually seen in children and are characterized by some unusual and noteworthy findings: intense systemic inflammation, some features usually seen in Kawasaki disease and vasoplegia [8]. The underlying mechanism of heart damage remains unclear as none of the patients had an endomyocardial biopsy.

The prolonged fever, along with high systemic inflammation, vasoplegia, myocardial involvement and some features of Kawasaki disease (atypical form) underlie a possible new spectrum of vasculitis and inflammatory diseases following SARS-CoV-2 infection rather than direct viral organ damage as previously reported in adults [11, 12]. Children with atypical Kawasaki disease, even with acute myocarditis, are known to be younger [13]. Also, in the Kawasaki disease shock syndrome acute myocarditis occurs in only 30% of the cases; platelets count, C-reactive protein and neutrophil count are lower [14, 15] than what we observe in our population.

Regarding the clinical aspects of Kawasaki disease, even atypical and the current early stage of the description of this new disease, we suggest a close follow-up of cardiac recovery and repeated ultrasound scan to detect the potential occurrence of coronary artery dilation.

## Conclusions

In children, the severe multisystem inflammatory emerging disease following SARS-CoV-2 infection highlights the variability and the large spectrum in host response to this novel virus and suggests that it may be one of the upcoming and unknown clinical post-infective complications of SARS-CoV-2 infection.

## Supplementary information

**Additional file 1.** Cases description, Table S1.

## Data Availability

Individual details of cases were provided in supplementary material.

## Abbreviations

COVID-19: Coronavirus disease 2019
CRP: C-reactive protein
PCR: Polymerase chain reaction
PCT: Procalcitonin
PICU: Pediatric intensive care unit
SARS-CoV-2: Severe acute respiratory syndrome-coronavirus-2
