# Supplementary material for: Acute myocarditis and multisystem inflammatory emerging disease following SARS-CoV-2 infection in critically ill children
Source: Ann Intensive Care. 2020 Jun 1;10:69. doi: 10.1186/s13613-020-00690-8 (PMC7266128; doi:10.1186/s13613-020-00690-8)
Supplement: Supplementary file 1 — Additional file 1. Cases description, Table S1. [file 13613_2020_690_MOESM1_ESM.docx]

|  | **Patient 1** | **Patient 2** | **Patient 3** | **Patient 4** | **Patient 5** | **Patient 6** | **Patient 7** | **Patient 8** | **Patient 9** | **Patient 10** |
| --- | --- | --- | --- | --- | --- | --- | --- | --- | --- | --- |
| **Age**, years | 8,7 | 15,0 | 12,7 | 6,0 | 8,3 | 11,7 | 8,5 | 6,8 | 3,9 | 10,4 |
| **Sex**, M/F | F | F | F | F | M | M | F | M | M | F |
| **Admission delay from first symptoms**, days | 6 | 6 | 6 | 6 | 7 | 6 | 6 | 7 | 6 | 5 |
| **PELOD-2 score at admission** | 22 | 11 | 11 | 20 | 10 | 10 | 10 | 10 | 21 | 10 |
| **Clinical description**  Fever  Abdominal pain  Skin rash  Conjunctivitis  Cheilitis  Adenitis (diameter > 1,5 cm)  Glasgow coma scale | Yes  Yes  Yes  No  No  No  11 | Yes  Yes  Yes  No  No  No  4 | Yes  Yes  No  No  No  No  15 | Yes  Yes  No  No  No  No  11 | Yes  Yes  No  No  No  Yes  13 | Yes  Yes  Yes  No  No  No  13 | Yes  Yes  Yes  No  Yes  No  15 | Yes  Yes  Yes  Yes  No  No  15 | Yes  Yes  Yes  No  No  No  15 | Yes  Yes  No  Yes  Yes  No  15 |
| Tachycardia  Arterial hypotension  Left ventricular ejection fraction, % | Yes  Yes  40 | Yes  Yes  30 | Yes  Yes  30 | Yes  Yes  35 | Yes  Yes  20 | Yes  Yes  45 | Yes  Yes  45 | Yes  Yes  35 | Yes  Yes  45 | Yes  Yes  30 |
| **Laboratory values***  Troponin, ng/mL  Brain Natriuretic Peptid, pmol/L  Lactate, mmol/L  Creatinine Clearance, mL/min/1,73 m^2^  Albumin, g/L  Sodium, mmol/L  Alanine aminotransferase, IU/L  Platelets count, per mm^3^  Neutrophil count, per mm^3^  Lymphocyte count, per mm^3^  C Reactive Protein, mg/L  Procalcitonin, ng/mL  Fibrinogen, g/L  **Inotropes and vasoactive drugs****  Epinephrine, µg/kg/min  Milrinone, µg/kg/min  Dobutamine, µg/kg/min  Norepinephrine, µg/kg/min  **Mechanical ventilation**  Non-invasive ventilation  Invasive ventilation  High flow nasal oxygen | 240  9180  5  51  23  128  57  271 000  34200  2500  450  40  7,9  0,1  0,3  0  1,2  No  Yes  No | 2360  4255  8  94  19  131  163  159 000  13680  610  256  NA  8  0,5  0,5  0  0,8  No  Yes  No | 372  2555  5,4  143  25,7  130  6  269 000  11300  1500  269  6,1  7,9  0,1  0,5  0  0  Yes  No  No | 654  19013  5,5  49  23,8  135  16  210 000  16800  1500  173  448  4,2  0,2  0,5  0  0  No  Yes  No | 959  14150  8,1  16  18,1  122  63  183 000  9660  900  351  54  6,2  0,4  0,3  0  0  No  Yes  No | 215  6378  3,4  116  17,3  131  75  165 000  18900  1400  458  NA  7,2  0,1  0  0  0,2  No  Yes  No | 361  2088  1,8  163  21,5  133  26  203 000  9900  1500  160  1,6  5,4  0,1  0  0  0  Yes  No  No | 287  11980  3  76  24,6  129  28  256 000  30500  900  454  347  8,1  0,2  0,6  5  0  Yes  No  No | 136  14657  1,3  69  20  129  22  213 000  1500  1800  208  66  5,8  0  0  5  0  Yes  No  No | 64  4256  2,4  51  18,2  133  101  403 000  9400  800  246  299  4,4  0  0,6  0  0  Yes  No  No |
| **SARS-COV-2 PCR**  Nasaopharyngeal swabs  Stools  **SARS-Cov-2 IgG/IgA** | -  -  +/+ | -  -  +/+ | -  -  +/+ | +  -  +/+ | -  +  +/+ | +  -  NA/NA | +  -  +/+ | -  -  +/+ | +  NA  +/+ | +  NA  +/+ |
| **Immune therapy**  Intravenous immunoglobulin  Corticosteroids  IL1 receptor antagonist  IL6 receptor antagonist | Yes  No  No  No | Yes  No  No  No | Yes  No  No  No | Yes  No  No  No | Yes  No  No  No | Yes  No  No  No | Yes  No  No  No | Yes  No  No  No | Yes  No  No  No | Yes  No  No  No |

|  | **Patient 11** | **Patient 12** | **Patient 13** | **Patient 14** | **Patient 15** | **Patient 16** | **Patient 17** | **Patient 18** | **Patient 19** | **Patient 20** |
| --- | --- | --- | --- | --- | --- | --- | --- | --- | --- | --- |
| **Age**, years | 7,2 | 11,8 | 11,1 | 4,6 | 8,7 | 8,1 | 2,9 | 8,4 | 12,6 | 11,4 |
| **Sex,** M/F | M | F | M | M | M | M | F | F | M | F |
| **Admission delay from first symptoms**, days | 5 | 10 | 1 | 7 | 3 | 4 | 4 | 6 | 6 | 2 |
| **PELOD-2 score at admission** | 10 | 11 | 21 | 10 | 11 | 11 | 11 | 10 | 10 | 10 |
| **Clinical description**  Fever  Abdominal pain  Skin rash  Conjunctivitis  Cheilitis  Adenitis (diameter > 1,5 cm)  Glasgow coma scale | Yes  Yes  No  No  Yes  Yes  15 | Yes  Yes  No  Yes  No  No  11 | Yes  Yes  No  Yes  No  No  15 | Yes  Yes  Yes  Yes  Yes  No  15 | Yes  Yes  No  No  No  No  15 | Yes  Yes  No  No  No  No  15 | Yes  Yes  No  No  No  No  12 | Yes  Yes  Yes  No  No  No  15 | Yes  Yes  No  Yes  Yes  No  15 | Yes  Yes  No  No  No  No  15 |
| Tachycardia  Arterial hypotension  Left ventricular ejection fraction, % | Yes  Yes  30 | Yes  Yes  35 | Yes  Yes  30 | Yes  Yes  45 | Yes  Yes  45 | Yes  Yes  40 | Yes  Yes  25 | Yes  Yes  25 | Yes  Yes  55 | Yes  Yes  33 |
| **Laboratory values***  Troponin, ng/mL  Brain Natriuretic Peptid, pmol/L  Lactate, mmol/L  Creatinine Clearance, mL/min/1,73 m^2^  Albumin, g/L  Sodium, mmol/L  Alanine aminotransferase, IU/L  Platelets count, per mm^3^  Neutrophil count, per mm^3^  Lymphocyte count, per mm^3^  C Reactive Protein, mg/L  Procalcitonin, ng/mL  Fibrinogen, g/L  **Inotropes and vasoactive drugs****  Epinephrine, µg/kg/min  Milrinone, µg/kg/min  Dobutamine, µg/kg/min  Norepinephrine, µg/kg/min  **Mechanical ventilation**  Non-invasive ventilation  Invasive ventilation  High flow nasal oxygen | 240  NA  3,7  119  20  128  33  93 000  6000  800  142  30  5,3  0,2  0,4  0  0  Yes  No  No | 211  NA  3,7  103  21,8  132  6  125 000  6100  600  344  52  5,7  0,1  0,4  0  0  No  Yes  Yes | 206  NA  2  65  20  129  11  398 000  13600  2200  393  27  8,6  0  0  15  0  Yes  Yes  No | 31  NA  1  170  22  132  16  129 000  7640  1680  94  15  3,9  0  0  0  0  No  No  No | 185  2225  5,2  121  NA  135  49  270 000  10010  750  213  NA  8,4  0  0  15  0  Yes  No  No | 530  2511  1,6  115  NA  133  32  177 000  11800  380  200  269  8,3  0  0  0  0  Yes  No  No | 250  NA  1,7  28  24  139  18  320 000  17900  7200  319  NA  9  0  0  10  0  No  No  No | 125  2305  1,7  168  20  130  10  211 000  14270  770  134  10  7  0,1  0  0  0  Yes  No  No | 4607  870  1,5  97  20,5  129  19  242 000  8260  510  227  9  NA  0  0  0  0  No  No  No | 545  2532  6  94  19,1  137  58  207 000  6400  430  456  64  8  0,3  0,3  0  0,3  Yes  Yes  No |
| **SARS-COV-2 PCR**  Nasopharyngeal swabs  Stools  **SARS-Cov-2 IgG/IgA** | +  +  +/+ | -  NA  +/+ | -  NA  +/+ | +  NA  +/+ | +  NA  NA/ NA | +  NA  NA/ NA | +  NA  NA/ NA | -  NA  NA/ NA | -  -  +/+ | -  -  +/+ |
| **Immune therapy**  Intravenous immunoglobulin  Corticosteroids  IL1 receptor antagonist  IL6 receptor antagonist | Yes  No  No  No | Yes  No  No  No | Yes  No  No  No | Yes  No  Yes  No | Yes  No  No  No | Yes  No  No  Yes | Yes  No  No  No | Yes  Yes  No  No | Yes  Yes  No  No | Yes  No  No  No |

F, female; IL, interleukine; M, male; NA, not available, PELOD-2, Pediatric Logistic Organ Dysfunction 2

* highest dosing during the PICU stay

** worst values within the first 24 hours of PICU stay
